# Tumor-targeting cell membrane-coated nanorings for magnetic-hyperthermia-induced tumor ablation

**DOI:** 10.1039/d3bm01141k

**Published:** 2023-09-26

**Authors:** Veena Vijayan, Aravindkumar Sundaram, Arathy Vasukutty, Rizia Bardhan, Saji Uthaman, In-Kyu Park

**Affiliations:** a Department of Biomedical Sciences, Chonnam National University Medical School 264 Seoyang-ro Hwasun Jeonnam 58128 Republic of Korea pik96@jnu.ac.kr; b Department of Chemical and Biological Engineering, Iowa State University Ames IA 50012 USA; c Nanovaccine Institute, Iowa State University Ames IA 50012 USA sajiuthaman@gmail.com

## Abstract

Magnetic hyperthermia has attracted considerable attention for efficient cancer therapy because of its noninvasive nature, deep tissue penetration, and minimal damage to healthy tissues. Herein, we have fused cancer cell membrane fragments with lipids and cloaked them on magnetic nanorings to form targeted Fe nanorings (TF) for tumor-targeted magnetic hyperthermia-induced tumor ablation. In our approach, cell membrane fragments from cancer cells were fused with lipids to form vesicles, which could efficiently encapsulate magnetic nanorings, thereby forming TF. We observed that TF have high tumor uptake *via* homotypic targeting, where cancer cells take up TF through membrane fusion. Under an external alternating magnetic field (AMF), TF accumulated in the tumors are heated, driving magnetic-hyperthermia-induced tumor cell death. Our *in vitro* studies show that self-targeting TF efficiently localized in cancer cells and induced cell death with an AMF, which was shown by a live/dead assay. Our findings demonstrate the potential of TF in tumor ablation, thereby making them promising and efficient nanosystems for tumor-targeted theranostics.

## Abstract 2

Tumor-targeting cell membrane-coated magnetic nanorings for enhanced tumor accumulation and magnetic-hyperthermia-induced tumor ablation.

## Graphical abstract

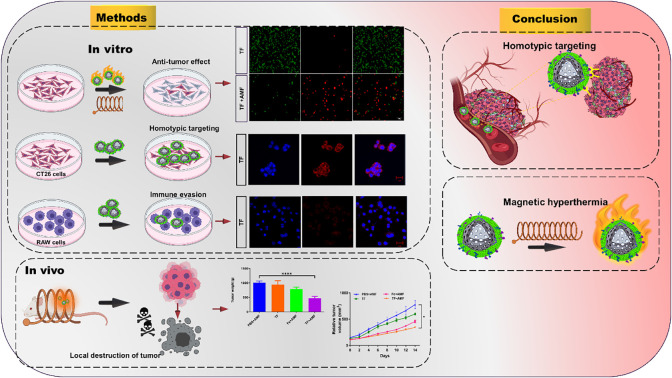

## 1. Introduction

In recent years, hyperthermia has emerged as an alternative to conventional solid tumor treatment strategies.^1,2^ It is a local anticancer therapy in which the tumor temperature rises above the physiologically optimal body temperature, damaging tumor tissues.^3^ In moderate hyperthermia, the heating temperature range is 40–46 °C, and a temperature above 46 °C is considered high. Temperatures above 60 °C are generally used for thermal ablation.^4^ Hyperthermia eliminates cancerous tissues with minimal damage to normal surrounding tissues and can be achieved using laser beams, microwaves, high-intensity focused ultrasound, and alternating magnetic fields (AMFs).^5–7^

Magnetic hyperthermia involves using an AMF and magnetic nanoparticles to heat tumors.^8,9^ It is non-invasive, and many clinical trials are investigating its safety and effectiveness for various cancers. Iron oxide nanoparticles are among the most used types of magnetic nanoparticles in magnetic hyperthermia clinical trials. In clinical trials, only intratumoral administration of iron oxide nanoparticles is available for patients and heat is generated under an AMF to inhibit tumor growth.^10^ Although they have been used for imaging and magnetic hyperthermia, they have low chemical stability under physiological conditions and poor tumor-targeting ability.^2,11^ Magnetic nanoparticles have been extensively used in cancer treatments as magnetic resonance contrast agents. They exhibit a darkening effect under static magnetic fields because of the short relaxation time of the protons (*T*_2_).^12–14^ Under an AMF, particles smaller than 10 nm generate heat *via* Néel's loss and Brownian motion, and larger particles generate heat *via* hysteresis loss. Heat generation can be controlled by varying the magnetic field intensity and frequency, and the temperature can be set above 42 °C to cause hyperthermia of cancer cells. Unlike normal cells, tumor cells are sensitive to the temperature range of 40–45 °C, and hence, the therapeutic temperature can be chosen in this range to avoid damage of normal tissues.^15^ During magnetic hyperthermia, the temperature of cancer cells reaches around 40–45 °C and causes apoptosis. Compared with other hyperthermia treatments, magnetic hyperthermia causes minimal damage to normal tissues as only magnetic particles are influenced by the magnetic field and damage deep-seated tumors.^9^

Magnetic iron oxide nanoparticles have many advantages, including high chemical stability, low toxicity, strong magnetic response, and ease of surface modification and functionalization. Moreover, they have been approved by the Food and Drug Administration (FDA) for biomedical use. They are used in computed tomography at high concentrations and magnetic resonance imaging (MRI) at low concentrations.^16,17^ Magnetic nanorings can have higher magnetic susceptibility and saturation magnetization than solid nanoparticles, which makes them more suitable for MRI and magnetic separation. They have been shown to have higher heating efficiency, owing to their hollow interior, than solid nanoparticles, and hence, they are capable of more efficient energy absorption and dissipation.^18,19^ They can be delivered to tumor sites *via* different methods, of which the intratumoral method is efficient for the effective heating of tumors; in the intravenous method, nanoparticles accumulate in the tumor through the enhanced permeation and retention effect because of the leaky vasculature of the tumor. Another delivery method involves using surface-modified nanoparticles to effectively target cancer cells.^13,20^ However, the intratumoral method is impractical for large tumors and metastasis models. For such tumors, an effective targeting moiety is required for nanoparticle accumulation in the tumor. Applying a magnetic field causes hyperthermia, which in turn induces tumor apoptosis. The preferential accumulation of nanoparticles in cancer^21^ cells under an AMF helps to selectively heat tumors without damaging the adjacent normal tissue.

Recreating certain biological functions in synthetic materials is a difficult task. This can be achieved by employing a biomimetic strategy in which natural materials can be used to endow nanoparticles with different biofunctional properties.^22–24^ The unique bio-properties of different cell types can be attributed to the membrane's complex antigenic profile, which helps identify and use these biomimetic membrane factors for different applications.^25,26^ Utilizing the active cell membrane and incorporating it onto nanoparticles offer the ability to replicate the surface antigens of the source cells used. Depending on the membrane antigens and membrane structure, the biomimetic nanoparticles can impart properties such as prolonged blood circulation, immune evasion, and ligand recognition, making them promising nanoplatforms for various biomedical applications.^27,28^ Cancer cell membranes have been reported to have homotypic targeting capability towards source cell lines and high internalization ability,^28,29^ and cancer cells show strong adhesion owing to specific membrane proteins on their surface.^29^ This property is exploited to coat cancer cell membranes onto nanoparticles for homologous targeting and stronger internalization of nanoparticles. This self-targeting property and the immune evasion property of cancer cell membranes make them suitable for surface decoration of nanoparticles for different applications.^30,31^

**Scheme 1:**
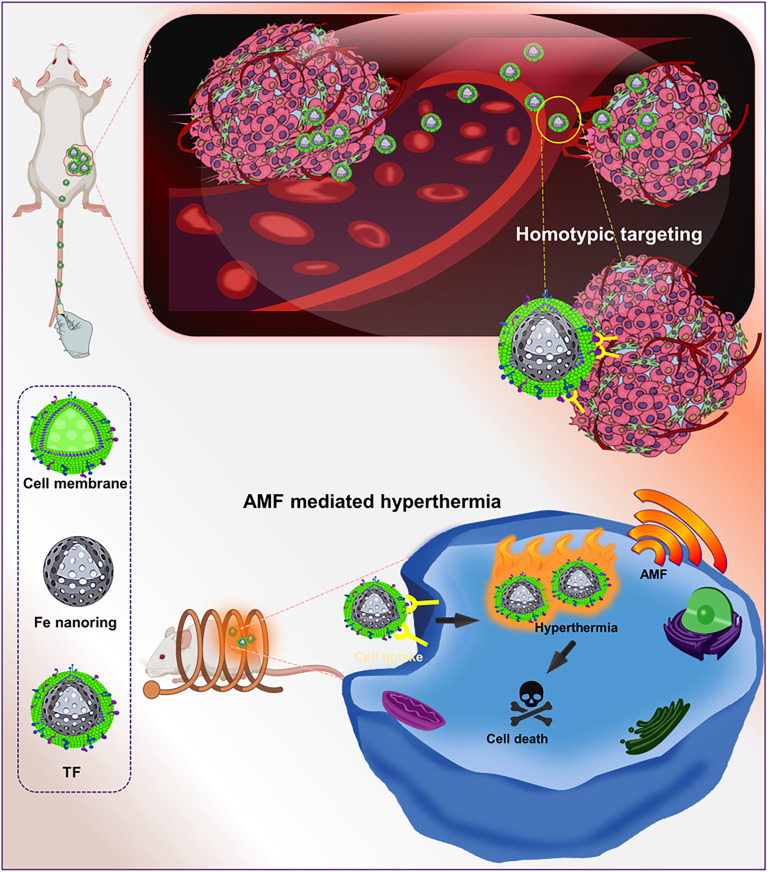
Schematic illustration of tumor-targeting cell membrane-coated nanorings for magnetic hyperthermia for tumor ablation.

In this study, we developed tumor targeting cell membrane-coated nanorings forming targeted Fe nanorings (TF) for achieving efficient magnetic hyperthermia and tumor growth inhibition. Water-soluble magnetic nanorings (Fe) were synthesized by the solvothermal process for AMF-mediated magnetic hyperthermia, and they were cloaked with a cancer cell–lipid hybrid membrane for effective tumor targeting (Scheme 1). The inherent homotypic targeting ability of TF was proved by cellular uptake, thereby aiding in immune evasion. The magnetic heating ability and cell death were demonstrated using a live/dead assay. The *in vivo* tumor ablation also explained the magnetic hyperthermic effect of TF paving the way for an enhanced tumor-targeted theragnostic nanoplatform.

## 2. Materials and methods

### 2.1 Reagents and cell lines

Ferric chloride hexahydrate (FeCl_3_·6H_2_O), polyethylene glycol (PEG-2000), anhydrous sodium acetate (NaOAc), and ethanolamine (ETA) were procured from Sigma-Aldrich, ethylene glycol (EG) was procured from Duksan (Gyeonggi, Korea), a WST-1 assay kit was obtained from SFC Probes (Gyeonggi, Korea) and 4′,6-diamidino-2-phenylindole (DAPI) was acquired from Thermo Fisher Scientific Korea Ltd (Seoul, Korea). Unless otherwise specified, all other chemicals used were of analytical grade and purchased from commercial suppliers. A dialysis membrane of molecular weight 6–8 kDa was purchased from Spectrum Laboratories, Inc. (CA, USA).

Mouse colorectal cancer (CT26) cells and mouse fibroblast cells (L929) were used for cell culture experiments. CT26 and L929 cells were cultured in Dulbecco's Modified Eagle's medium (DMEM) acquired from Welgene (Fresh Media™, South Korea) with 10% fetal bovine serum and 1% antibiotics as supplements. Cells were kept at 37 °C in a humidified environment with 5% CO_2_. Cell lysis buffer (10×) and proteinase inhibitor phenylmethanesulfonyl fluoride (PMSF) were purchased from Cell Signaling Technology, Danvers, MA, USA.

### 2.2. Magnetic nanoring synthesis

Magnetic nanorings were synthesized using a solvothermal method by dissolving 1 g of FeCl_3_·6H_2_O in a solvent containing 30 mL of EG and 10 mL of ETA. To this solution, 1 g of PEG-2000 and 4 g of sodium acetate were added, and then this solution was heated for 12 h at 200 °C.^32^ The obtained particles were magnetically separated, washed multiple times with ethanol and deionized water, and lyophilized.

### 2.3. TF preparation

First, cancer cell membrane fragments were obtained using an ultracentrifugation technique.^33,34^ CT26 cells were incubated in T175 flasks, detached using trypsin, and a pellet was collected by centrifugation at 1200*g*, which was washed with phosphate-buffered saline (PBS) and centrifuged again at the above speed. The as-obtained pellet was resuspended in PBS containing cell lysis buffer and PMSF and incubated in ice for 15 min. Subsequently, the cells in the solution were lysed by sonication, and the solution was subjected to centrifugation at 20 000*g* for 20 min at 4 °C. The pellet was discarded, the supernatant solution was centrifuged at 100 000*g* for one hour at 4 °C to obtain a pellet, and the membrane fragments were lyophilized and stored at −80 °C for further use. The membrane fragments were dispersed in PBS before use.

To prepare TF, the thin film hydration method was used. In brief, dipalmitoyl-*sn*-glycerol-3-phosphocholine and cholesterol lipids (Avanti Polar Lipids, Alabama, USA) dissolved in methanol were dried to form a thin film in a rotary evaporator, which was rehydrated at 60 °C for 7 min with continuous stirring. The resulting solution was mixed with hollow iron oxide nanoparticles and membrane fragments in a 1 : 2 weight ratio, sonicated using a probe sonicator (amplitude 23%) for 7 min, and subjected to dialysis using a 6–8 kDa dialysis membrane against water for four hours. Using an Avanti extruder, the solution was transferred to a syringe and extruded successively through 1 μm, 400 nm, and 200 nm polycarbonate membrane filters.

### 2.4. Characterization of TF

Field emission transmission electron microscopy (FE-TEM; JEM-2100F, JEOL USA Inc., Peabody, MA, USA) was used to analyze the TF morphology. The iron content of the magnetic nanorings was analyzed by thermogravimetric analysis (TGA) and compared with that of oleic acid-capped SPION. High-resolution (HR) X-ray photoelectron spectroscopy (XPS) was used to investigate the atomic composition of hollow iron oxide nanoparticles. The surface area and adsorption/desorption curve of nanorings were determined by the Brunauer–Emmett–Teller (BET) method. The magnetic properties of Fe nanorings were determined using a vibrating sample magnetometer (7400 series, Lake Shore Cryotronics).

### 2.5. Characterization of cancer cell membrane proteins

Western blot was used to characterize the cancer cell membrane proteins. A bicinchoninic acid assay kit (BCA; Thermo Scientific, Waltham, MA, USA) was used to determine the protein concentrations following the manufacturer's instructions, and protein samples were separated based on molecular weight using 10% sodium dodecyl sulfate-polyacrylamide gel electrophoresis analysis and were transferred to methanol-pre-activated polyvinylidene difluoride (PVDF) membranes. Furthermore, 5% skimmed milk was used to block the PVDF membranes, which were incubated overnight with primary antibodies (Na^+^/K^+^ ATPase, pan-cadherin) and anti-β-actin antibodies (Santa Cruz Biotechnology, Dallas, Texas, USA) at 4 °C. They were further incubated with secondary horseradish peroxidase-conjugated antibodies, and the blots were imaged using iBright (Thermo Fisher Scientific Ltd, Seoul, Korea).

### 2.6. *T*_2_ relaxation time of magnetic nanorings

The *T*_2_ relaxation time was determined by serial dilution of nanoparticles from 0.25 mM Fe and MRI experiments were conducted using a 3T clinical MRI instrument. For relaxivity measurement, *T*_2_-weighted scans were obtained with a time of repetition of 2400 ms and a time of echo in the range from 20 to 200 ms. Relaxivity was calculated by least-squares curve fitting of the relaxation time *versus* iron concentration plot.

### 2.7. Cell viability

The cytocompatibility of TF was evaluated using CT26 cells. Briefly, 1 × 10^4^ cells were seeded in a 96-well plate and incubated overnight at 37 °C with 5% carbon dioxide (CO_2_). The cell medium was then removed, different concentrations of samples were added, and the plates were maintained at 37 °C for 24 h. The cell viability profile was assessed using the WST-1 assay by following the manufacturer's protocol.

To check if the nanoparticles were cytocompatible, we used L929 cells. Briefly, 1 × 10^4^ cells were seeded in a 96-well plate and incubated at 37 °C with 5% carbon dioxide (CO_2_). The culture medium was removed, different concentrations of samples were added, and further incubated for 24 h at 37 °C. The cell viability profile was analyzed using the WST-1 assay by following the manufacturer's protocol.

### 2.8. Cellular uptake

The TF uptake by CT26 cells was evaluated using the Prussian blue technique. Briefly, 3 × 10^5^ CT26 cells were seeded in an eight-well chamber slide (Lab-Tek II, Utah, USA) and incubated for 24 h at 37 °C with 5% CO_2_. Then, the medium was removed and washed with DPBS twice before adding 50 μg mL^−1^ TF and Fe nanorings to CT26 cells and kept for four hours. It was washed with DPBS several times to remove unbound samples and later fixed with 4% paraformaldehyde (PFA), incubated for some time, and washed again with DPBS. The Prussian blue staining solution was added, incubated for 20 min, and then washed with DPBS. Subsequently, nuclear fast red was added as a counter stain for 5 min, and then the cells were rinsed with DPBS to remove the staining solution. The cells were washed with 70% and 100% ethanol and mounted on a glass slide using PEH mounting solution. The cells were imaged using an inverted light microscope.

### 2.9. *In vitro* homotypic targeting and immune evasion ability of TF

A confocal microscope was used to examine the homotypic targeting ability of TF in CT26 cells. Briefly, 2 × 10^4^ CT26 cells were grown in an eight-well chamber slide for 16 h at 37 °C with 5% CO_2_. The media were removed, and fresh media containing samples were added. DiD was the model fluorescent dye used for loading TF into the cancer cell membrane. The cells were incubated with DiD, RBC membrane vesicles, and TF-DiD for four hours, and the medium was then aspirated, rinsed with DPBS thrice, and fixed with 4% PFA, and DAPI was added for staining the nucleus. For the *in vitro* immune escape study, we seeded macrophage RAW 264.4 cells in a similar way to that mentioned above. The media were replaced with fresh media containing samples, incubated for 4 h, rinsed with DPBS, and fixed with 4% PFA, and the nucleus was stained with DAPI.

### 2.10. *In vitro* magnetic hyperthermia study

CT26 cells were used for performing *in vitro* magnetic hyperthermia experiments. First, 2 × 10^5^ cells were seeded in a 10 cm round dish and cultured overnight. The media were replaced with fresh media containing different samples (200 μg mL^−1^ TF and magnetic Fe nanorings), incubated for 4 h, and subjected to an AMF (H.F Induction Heater, Insung Heavy Electronics, Busan, Korea) for 10 min (120 V, 303 kHz). Live/dead staining was used to assess the hyperthermic ability of TF with and without an AMF, and it was performed by mixing ethidium homodimer-1 and calcein-AM in DPBS and incubating them with cells for 15 min. The cells were rinsed with DPBS and imaged using a ZOE fluorescent cell imager (Bio-Rad, USA). The number of red and green cells was counted from the images captured.

### 2.11. *In vivo* antitumor therapy

All mice experiments were conducted in agreement with the Chonnam National University Hospital and Chonnam National University Medical School institutional laws by following approved guidelines (approval number: CNU IACUC-H-2022-28). A subcutaneous CT26 tumor model was formed by hypodermically injecting 100 μL of 1 × 10^6^ cells on the right flank of mice. Once the tumor volume reached 100–130 mm^3^, mice were randomly sorted and grouped as PBS + AMF, Fe + AMF, TF, and TF + AMF (*n* = 4). Samples were injected intravenously and mice were subjected to AMF treatment 24 h after injection once every four days three times. For inducing hyperthermia, tumor-bearing mice were placed inside a plastic holder such that the tumor was positioned at the center of the copper coil of the AMF machine used to generate a magnetic field. The frequency of the machine was 303 kHz, the voltage was 120 V, and the power intensity was 16 for 10 min. The tumor volume was calculated using *V* = 0.5(length × width^2^). The tumor volume and the body weight of the mice were determined every two days until sacrifice.

### 2.12. Hematoxylin and eosin staining

15 days post-injection, tumors were excised, fixed with 10% neutral formaldehyde, embedded in paraffin, and sectioned. The sections were stained with H&E and observed under an inverted microscope.

### 2.13. Immunohistochemistry staining

Immunohistochemistry analysis of tumor sections was performed using the immunofluorescence technique, in which paraffin sections were rehydrated using a xylene/ethanol gradient and further kept at 100° C for 20 min in sodium citrate buffer for antigen retrieval. The sections were further blocked with 2.5% bovine serum albumin in PBS, incubated with Hsp 70 primary antibodies (Abcam) overnight at 4° C, washed, and then incubated with Flamma 488-conjugated secondary antibodies (BioActs). DAPI was used as a counter stain and the images were quantified using ImageJ software. The percentage of positive Hsp 70 cells with respect to positive DAPI cells was calculated and plotted as quantified data.

### 2.14. Statistics

Results are expressed as mean ± standard deviation and examined using GraphPad Prism software (San Diego, CA, USA). Statistical analysis was done using one-way ANOVA.

## 3. Results

### 3.1. Synthesis and characterization of TF

**Fig. 1:**
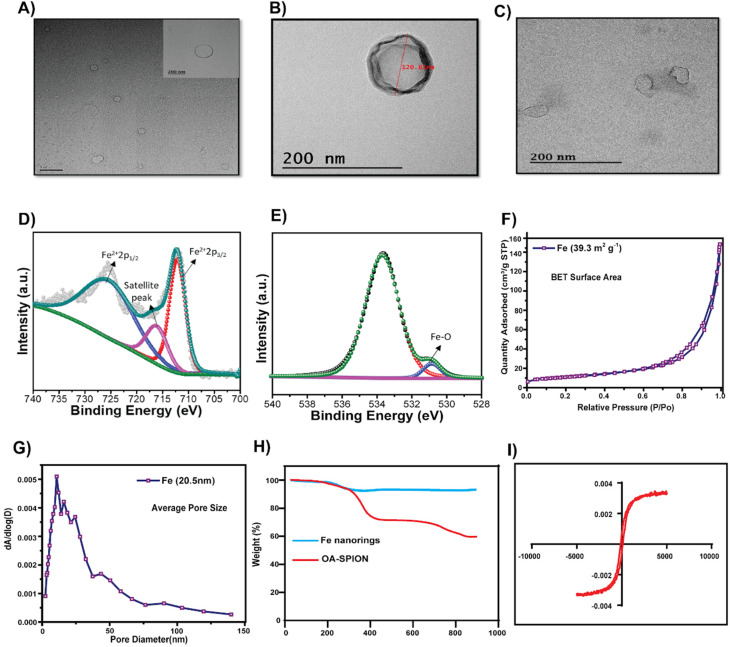
TEM images of (A) Fe (scale bar 1 μm; inset 200 nm), (B) TF and (C) cancer cell membrane. (D) XPS plot showing the deconvoluted spectrum of Fe 2p multiple peaks and (E) the peak at 531 eV represents the bonding between O and Fe in the synthesized particles, and the peak at 534 is related to surface oxidation (FeO_*x*_). (F) BET curves of Fe nanorings, (G) a BJH plot showing the distribution of pores, (H) results of TGA analysis of Fe and comparison with that of oleic acid-capped SPIONS, and (I) the hysteresis loop of Fe.

Magnetic nanorings (Fe) were synthesized using a solvothermal method.^32^ Hollow structures were formed through intense coordination between the amide of ethanolamine and Fe^3+^; ETA acted as a structure-controlling agent, and EG was used as a reducing agent. TEM showed that the particle size of Fe was around 110 nm (Fig. 1A). The hydrodynamic size of TF was 207 ± 29 nm with a surface charge of −30.6 ± 1.7 mV and that of the Fe nanorings was 170.27 ± 15.2 nm with a surface charge of 23.86 ± 5.4 mV. The negative surface charge of TF is attributed to the membrane coating on TF and the slight increase in the hydrodynamic size proves the successful coating of the membrane onto the nanorings. Fig. 1D and E show the XPS analysis data for Fe nanorings. Fig. 1D shows the deconvoluted spectrum of Fe 2p multiple peaks.

**Fig. 2:**
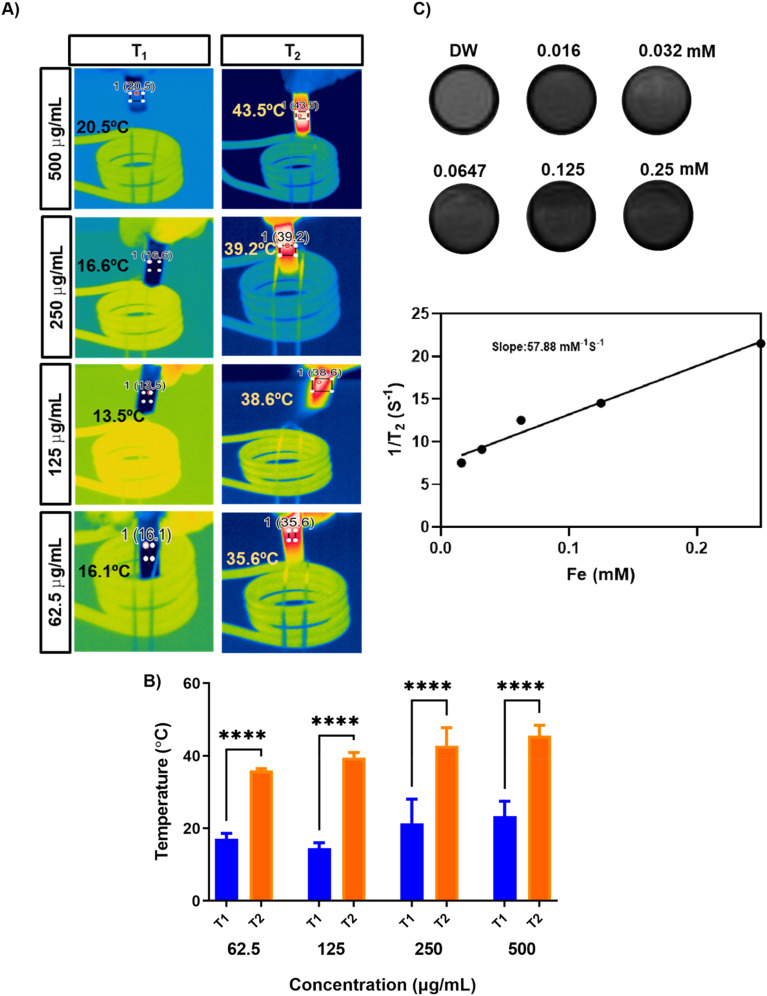
(A) AMF-induced heating of different concentrations of Fe nanoparticles. (B) Temperature increase profile of different concentrations of Fe. (C) *T*_2_-weighted MR images for different concentrations of Fe and its relaxation rate (1/*T*_2_).

The peaks at 712 and 725 eV are related to Fe^2+^ 2p_1/2_ and Fe^2+^ 2p_3/2_, respectively, and the weak peak centered at 717 eV represents the satellite peak.^35^ The peak at 531 eV represents the bonding between O and Fe in the synthesized particles, and the peak at 534 eV is related to surface oxidation (FeO_*x*_) (Fig. 1D).^36–38^ BET analysis (Fig. 1F and G) showed that the particles were porous, with an average pore size of around 20.5 nm and a BET surface area of 39.3 m^2^ g^−1^. Fig. 1H shows the results of the TGA analysis of Fe and oleic acid-capped SPION, indicating that the iron content in Fe nanorings is 92.7% compared with that of SPION. Fig. 1I shows the magnetization property of Fe; a magnetic hysteresis curve can be observed. To examine the AMF-induced heating ability, we subjected Fe nanoparticles to an AMF for 10 min at 100 V and found their heating efficiency to be concentration-dependent (Fig. 2A). The temperature increase for different concentrations of Fe was found to be significantly high even at low concentrations (Fig. 2B). To evaluate the *T*_2_ enhancing ability of Fe, a *T*_2_-weighted MRI was used to image aqueous solutions of different iron concentrations (Fig. 2C). *T*_2_ relaxivity measurements showed a direct relationship between the iron concentration and 1/*T*_2_ values. The relaxivity *R*_2_ was found to be 57.88 mM^−1^ s^−1^, which indicated the magnetic behavior of Fe, which enables its use as a contrast agent for *T*_2_ contrast imaging.

### 3.2. Characterization of cancer cell membrane proteins

**Fig. 3:**
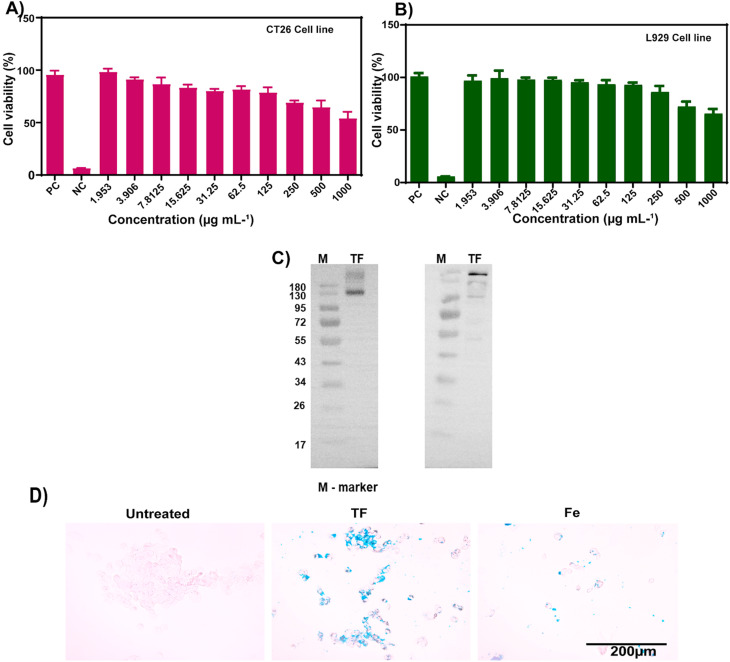
Cytocompatibility of TF for (A) CT26 cells and (B) L929 cells (triplicate). (C) Western blot confirming the presence of membrane proteins Na^+^/K^+^ ATPase and pan-cadherin in TF. (D) Uptake of TF and Fe nanoparticles by CT26 cells, examined by Prussian blue staining.

Purified cell membranes were first obtained to cloak membranes of CT26 cells onto hollow iron oxide nanoparticles. Using CT26 cell lines as source cells, we performed membrane derivation by emptying the intracellular contents of cells through cell lysis and sonication and then conducting ultracentrifugation. The collected cell membranes were then coated onto the core nanoparticles using a series of extrusions. After the fusion of membranes and nanoparticles, the membrane coating of the final TF was visualized using FE-TEM, and it was found to be around 120 nm in size (Fig. 1B). The protein content of the isolated cell membrane was examined by analyzing the cancer cell membrane proteins by western blot. The presence of Na^+^/K^+^ ATPase and pan-cadherin was confirmed in the isolated membrane. Thus, the membrane proteins from the source cells were successfully retained (Fig. 3C).

### 3.3. Cell viability and cellular uptake

The cytocompatibility of TF was examined for two cell lines (Fig. 3A and B). CT26 and L929 cell lines were treated with TF to check if the particles had any toxic effects on the cell lines. L929 is used as a control cell line in cytocompatibility studies. It showed minimal toxicity, and the percentage of viable cells was around 70 ± 5%, for a higher concentration of 1 mg mL^−1^ (Fig. 3A and B). In CT26 cells, the percentage was about 55 ± 8% for 1 mg mL^−1^ TF incubation for 24 h. Cellular uptake was evaluated by Prussian blue staining. The uptake of both TF and Fe was observed in the CT26 cell line (Fig. 3D). However, the uptake of TF was higher than that of Fe and that in control groups. This higher uptake is attributed to the homotypic targeting of the membrane obtained from its source cell line.

### 3.4. *In vitro* homotypic targeting and immune evasion ability of TF

**Fig. 4:**
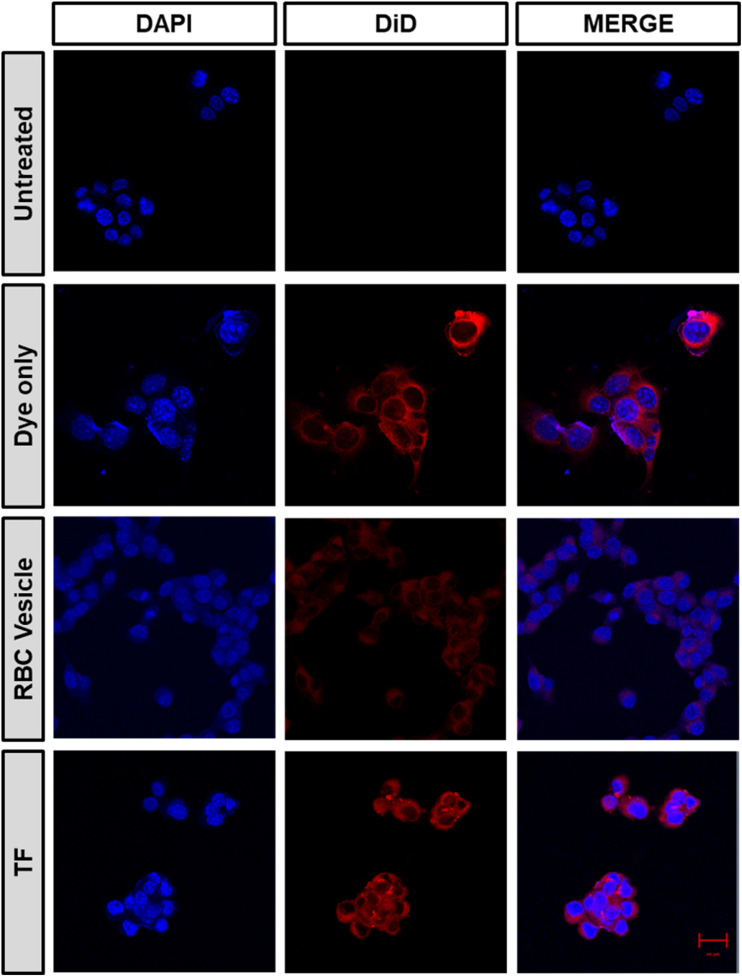
Confocal microscopy images confirming the homotypic targeting effect of TF, obtained after incubating CT26 cells with DiD dye-loaded TF, RBC-membrane vesicles, and only dye (scale bar 20 μm).

**Fig. 5:**
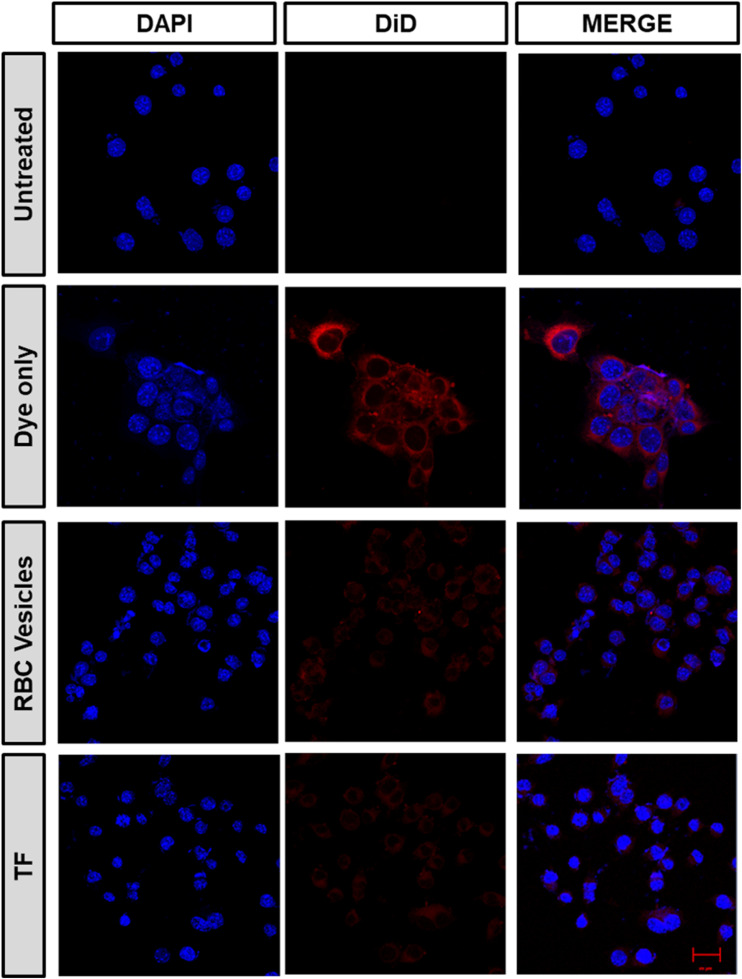
Immune evasion ability of TF obtained after incubating CT26 cells with dye-loaded TF, RBC-membrane vesicles, and dye only (scale bar 20 μm).

**Fig. 6:**
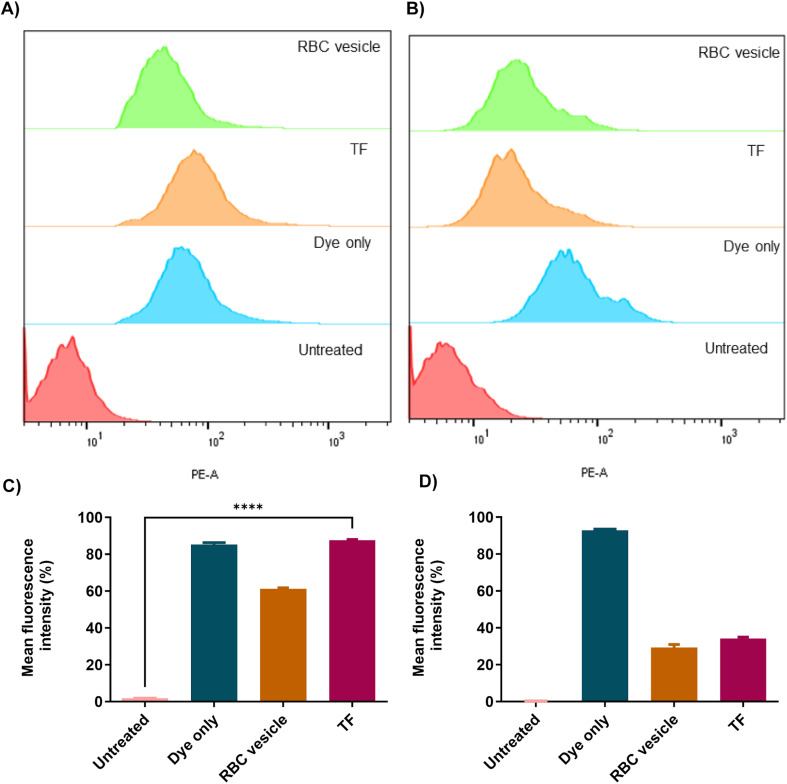
FACS analysis showing (A) the homotypic targeting efficiency of different groups in CT26 cells and its quantification is shown in (C). FACS analysis showing the immune evasion capability of different groups in RAW cells (B) and its quantification is shown in (D).

To demonstrate the ability of the TF to homotypically target their source cancer cell line, CT26 cell membrane vesicles were obtained and loaded with a fluorescent dye, DiD. Using confocal microscopy, we found that the fluorescent dye loaded membrane vesicles showed a higher uptake in CT26 cell lines than other control groups (Fig. 4). To examine the immune evasion ability of TF, we incubated dye-loaded membrane vesicles with macrophage cells RAW 264.7 and observed them under a confocal microscope (Fig. 5). There was a dramatic decrease in the uptake of TF by macrophage cells, which indicated that cancer cell membranes showed immune evasion ability like normal cells. FACS analysis was done to validate the homotypic targeting and immune evasion properties of membrane-coated nanorings as shown in Fig. 6. In Fig. 6A, TF groups show higher uptake in CT-26 cells compared to that in untreated cells. The dye-only group showed a similar effect because of the hydrophobic nature of the dye. Meanwhile, the FACS data for immune evasion also showed similar effects to those seen in confocal images. TF and RBC vesicles showed decreased cell uptake in RAW cells and the uptake of dye in both CT-26 and RAW cells was similar.

### 3.5. *In vitro* magnetic hyperthermia study

**Fig. 7:**
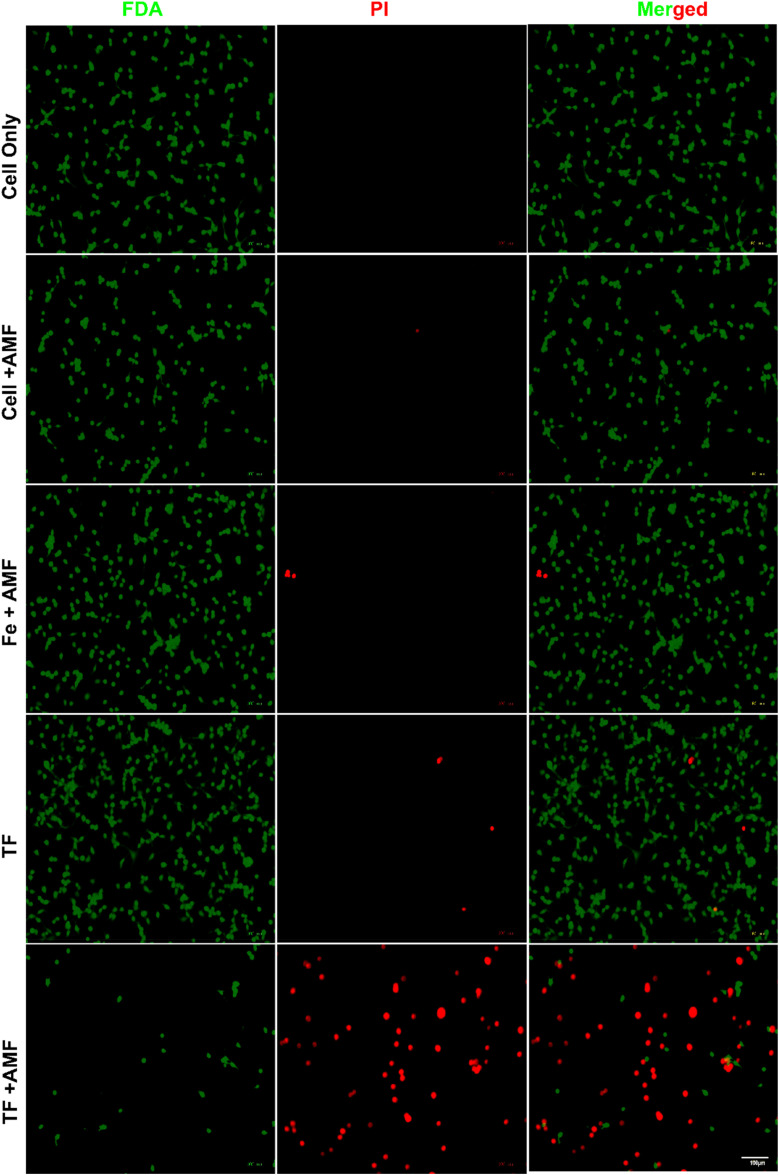
*In vitro* magnetic hyperthermic effect shown by live/dead staining of different nanoparticles in CT26 cells after AMF irradiation for 10 min at 120 V (scale bar 100 μm).

The magnetic field-mediated hyperthermia of TF for cancer application was studied using live/dead staining (Fig. 7). CT26 cells were incubated with AMF, Fe + AMF, TF, and TF + AMF for comparison. Cells without any treatment were considered as control. Untreated cells with and without AMF did not show any cell death; however, cells with Fe + AMF and TF showed negligible death compared to those with TF + AMF, possibly due to the membrane coating. The membrane coating resulted in higher uptake and heating efficiency, resulting in cell death. The live/dead staining study result could be considered a confirmation of the effective uptake of TF by the cancer cells. Selective tumor targeting and higher uptake result in effective hyperthermia.

### 3.6. *In vivo* tumor targeting ability and antitumor therapy

**Fig. 8:**
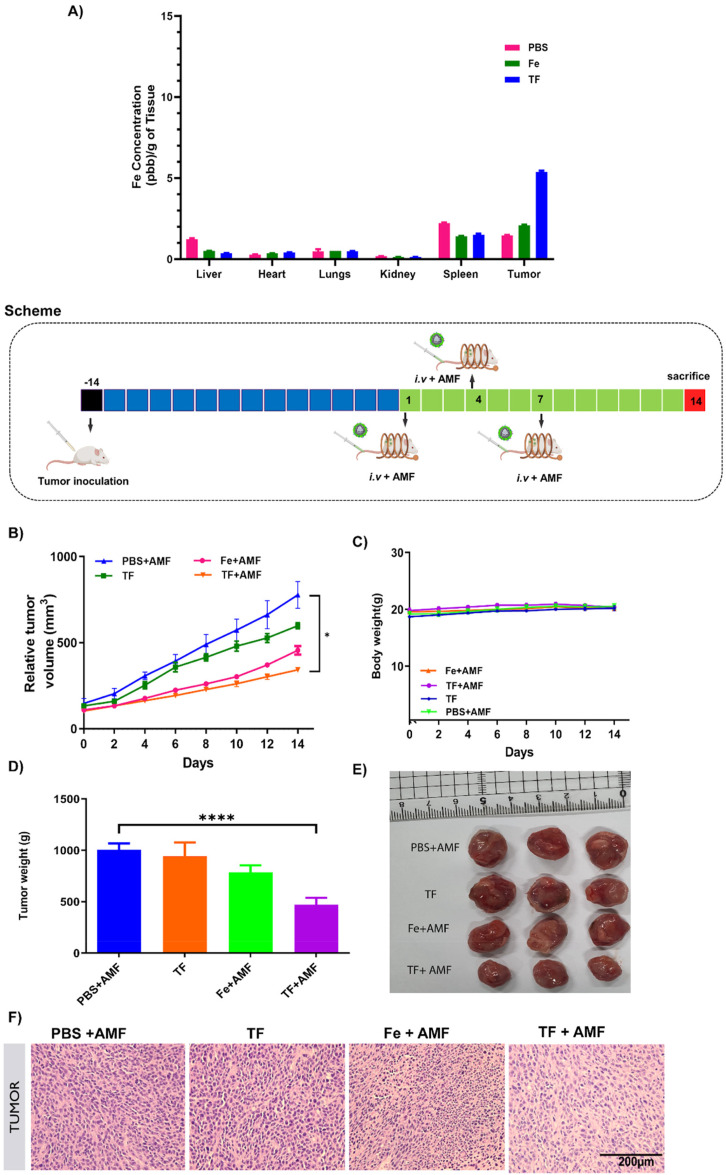
Antitumor effect of nanoparticles *in vivo*. (A) Fe content in all major organs and tumor tissues in mice 24 h post injection of PBS, Fe and TF. Schematic illustration of treatment, tumor volume (B), body weight (C), tumor weight (D), and tumor images (E) of mice treated with PBS + AMF, TF, Fe + AMF, and TF + AMF. (F) H&E images of tumor sections.

The *in vivo* tumor targeting ability of membrane-coated nanorings was checked by ICP-MS analysis. The Fe content in each group was measured in all major organs and tumor tissues after treatment for 24 h. The tumor tissues of the TF group showed increased Fe content compared to those in the Fe and PBS groups (Fig. 8A), validating the enhanced tumor targeting ability of membrane-coated nanorings.

The suitability of TF for AMF-mediated hyperthermia *in vivo* was assessed using CT26-bearing tumor mice. When the tumor reached 100 mm^3^, the mice were divided into PBS + AMF, Fe + AMF, TF, and TF + AMF groups. After intravenous sample injection, AMF was applied to all mice based on the treatment, and the tumor volume was measured every other day for up to 14 days. Based on the tumor volume, it was observed that the TF + AMF group showed significant tumor inhibition compared with the PBS + AMF control group (Fig. 8). The suppression of tumor growth might be due to magnetic hyperthermia and the effective tumor-targeting property of TF. Fig. 8 shows a significant decrease in the tumor weight in the TF + AMF group compared with the PBS + AMF group, which is similar to the tumor volume trend obtained. This indicates the importance of the tumor-targeted nano-delivery vehicle, which was efficiently obtained from the cancer cell membrane due to its inherent tumor targeting ability that resulted in high accumulation of Fe nanorings in the tumor.^39^ It was also achieved due to the heating ability of Fe nanorings under an AMF, thereby increasing the tumor temperature and facilitating efficient tumor ablation.

**Fig. 9:**
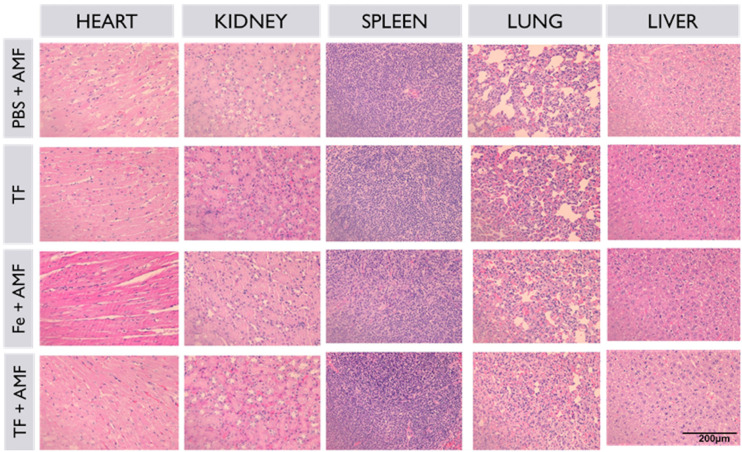
Histopathological analysis of major organs and tumors of all groups collected after the treatment period.

The *in vivo* toxicity of TF and other groups was investigated with H&E staining (Fig. 9). All major organs, such as the kidneys, liver, heart, lungs, and spleen, and tumors of all groups were used for H&E staining. No morphological changes were observed in any group after sample treatment, suggesting that the nanoparticles were biocompatible and non-toxic.

**Fig. 10:**
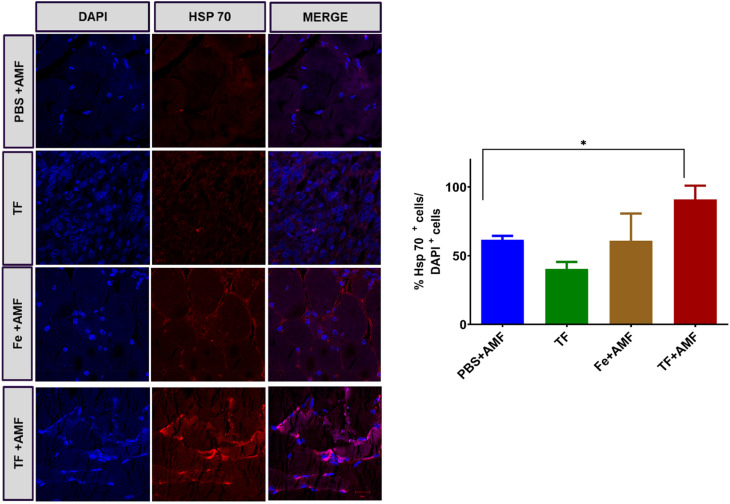
Immunohistochemistry staining of Hsp 70 produced in tumor tissues of PBS + AMF, Fe + AMF, TF, and TF + AMF groups (scale 20 μm) and the quantification of positive Hsp 70 cells.

Magnetic hyperthermia induces the production of heat shock proteins in tissues, and this was verified by immunohistochemistry staining of Hsp 70 protein in different groups. As shown in Fig. 10, increased fluorescence was observed in the TF + AMF group compared to that in PBS + AMF, TF and Fe + AMF groups. It is also noticeable that the TF group without any AMF irradiation showed decreased production of Hsp 70.

## 4. Conclusion

We developed tumor targeting cell membrane-cloaked nanorings for efficient magnetic hyperthermia. The biomimetic nanosystem comprises TF formulated with the cancer cell membrane, lipids, and Fe nanorings. Tumor reduction in mice treated with TF + AMF indicated that the TF successfully targeted homotypic tumors, which could be due to the successful translocation of membrane proteins required for self-adhesion that is helpful for immune evasion from macrophages. This self-targeting property of TF towards homotypic tumor cells and their immune evasion capability were observed in *in vitro* studies. Furthermore, magnetic heating was found to induce tumor apoptosis in *in vitro* and *in vivo* studies. Finally, coating the membrane onto the core nanoparticles resulted in excellent self-targeting, higher internalization, and favorable immune evasion ability of the source cells *in vitro*. This nature-inspired biomimetic strategy could be utilized for self-targeting tumor recognition in cancer theranostics using membranes from source tumors.^40,41^

## Author contributions

All authors contributed to the writing of this manuscript, and all contributors approved the final version of the manuscript.

## Conflicts of interest

The authors have no competing interests relevant to this study to disclose.

## Supplementary Material
